# CAAF-ResUNet: Adaptive Attention Fusion with Boundary-Aware Loss for Lung Nodule Segmentation

**DOI:** 10.3390/medicina61071126

**Published:** 2025-06-22

**Authors:** Thang Quoc Pham, Thai Hoang Le, Khai Dinh Lai, Dat Quoc Ngo, Tan Van Pham, Quang Hong Hua, Khang Quang Le, Huyen Duy Mai Le, Tuyen Ngoc Lam Nguyen

**Affiliations:** 1 Department of Pathology, University of Medicine and Pharmacy at Ho Chi Minh City, Ho Chi Minh City 700000, Vietnam; phamquocthang@ump.edu.vn (T.Q.P.); ngoquocdat@ump.edu.vn (D.Q.N.); pvtan.ntgpb21@ump.edu.vn (T.V.P.); huahongquang@ump.edu.vn (Q.H.H.); khanglequang@ump.edu.vn (K.Q.L.); 2 Faculty of Information Technology, University of Science, Ho Chi Minh City 700000, Vietnam; laidinhkhai@sgu.edu.vn; 3 Faculty of Information Technology, Vietnam National University, Ho Chi Minh City 700000, Vietnam; 4 Department of Diagnostic Imaging, University Medical Center, Ho Chi Minh City 700000, Vietnam; huyen.ldm@umc.edu.vn (H.D.M.L.); tuyen.nnl@umc.edu.vn (T.N.L.N.)

**Keywords:** lung nodule segmentation, adaptive attention controller, adaptive attention fusion, boundary-aware loss functions

## Abstract

*Background and Objectives*: The accurate segmentation of pulmonary nodules in computed tomography (CT) remains a critical yet challenging task due to variations in nodule size, shape, and boundary ambiguity. This study proposes CAAF-ResUNet (Context-Aware Adaptive Attention Fusion ResUNet), a novel deep learning model designed to address these challenges through adaptive feature fusion and edge-sensitive learning. *Materials and Methods*: Central to our approach is the Adaptive Attention Controller (AAC), which dynamically adjusts the contribution of channel and position attention based on contextual features in each input. To further enhance boundary localization, we incorporate three complementary boundary-aware loss functions: Sobel, Laplacian, and Hausdorff. *Results*: An extensive evaluation of two benchmark datasets demonstrates the superiority of the proposed model, achieving Dice scores of 90.88% on LUNA16 and 85.92% on LIDC-IDRI, both exceeding prior state-of-the-art methods. A clinical validation of a dataset comprising 804 CT slices from 35 patients at the University Medical Center of Ho Chi Minh City confirmed the model’s practical reliability, yielding a Dice score of 95.34% and a notably low Miss Rate of 4.60% under the Hausdorff loss configuration. *Conclusions*: These results establish CAAF-ResUNet as a robust and clinically viable solution for pulmonary nodule segmentation, offering enhanced boundary precision and minimized false negatives, two critical properties in early-stage lung cancer diagnosis and radiological decision support.

## 1. Introduction

Lung cancer remains a significant global health challenge, accounting for substantial morbidity and mortality due to its typically late-stage diagnosis [1]. The accurate segmentation and identification of lung nodules on computed tomography (CT) scans are essential for timely intervention, improved prognosis, and effective treatment planning. Despite substantial progress in computational imaging and deep learning approaches, precisely delineating lung nodules continues to pose substantial challenges because of their variability in shape, subtle textural differences, small sizes, and indistinct boundaries, complicating automated segmentation and often leading to high false- positive rates in clinical practice.

Deep-learning-based segmentation methods, particularly convolutional neural networks (CNNs) augmented with attention mechanisms, have significantly advanced medical imaging analysis, demonstrating improved accuracy by selectively emphasizing clinically critical regions. Nevertheless, conventional attention approaches commonly utilize fixed or predefined attention weighting schemes, limiting their flexibility and robustness across diverse medical imaging scenarios, especially those characterized by complex anatomical structures and heterogeneous nodule presentations.

To overcome these limitations and enhance segmentation accuracy, this study proposes the Channel-wise Adaptive Attention Fusion Network (CAAF-Net), an advanced segmentation architecture that introduces an improved Adaptive Attention Controller (AAC). Our AAC module innovatively employs adaptive, data-driven learning to generate optimal fusion weights dynamically, effectively leveraging complementary channel-wise and positional attention characteristics. This Adaptive Attention Fusion (AAF) approach not only enhances segmentation performance but also notably improves the model’s adaptability and generalization capability across different nodule morphologies and imaging conditions.

Moreover, recognizing the critical importance of precise boundary delineation in clinical diagnostics, our study introduces comprehensive boundary-aware loss functions, integrating Dice with Sobel, Laplacian, and Hausdorff distances. This boundary-centric strategy systematically enhances the model’s sensitivity to subtle boundary distinctions, substantially elevating its practical utility in clinical workflows.

Key contributions of our research include the following:

- A novel CAAF-ResUNet framework integrating an improved Adaptive Attention Controller (AAC), which dynamically generates attention fusion weights, significantly enhancing segmentation precision.
- Advanced boundary-aware loss strategies explicitly designed to improve segmentation accuracy at challenging nodule boundaries, effectively addressing a longstanding limitation of existing segmentation approaches.
- Extensive validation using benchmark datasets (LUNA16 and LIDC-IDRI) and clinical data comprising 35 real-world patient CT scans provided by the University Medical Center Ho Chi Minh City, confirming the robustness, clinical relevance, and adaptability of the proposed model.

### 1.1. Literature Review

Lung nodule segmentation in CT scans is a foundational task in medical imaging and plays a pivotal role in the early detection of lung cancer. Over the past decade, numerous segmentation architectures have been proposed, with deep learning-based approaches, especially encoder–decoder models like U-Net, establishing a strong performance baseline.

As segmentation accuracy has improved, research attention has shifted toward enhancing feature fusion mechanisms and improving contextual understanding, particularly through attention-based modules. Among the many techniques explored, three model families have become dominant: (i) U-Net and its variants, (ii) attention-enhanced models, and (iii) Transformer-based architectures. These form the basis of our review.

While many recent models have introduced attention mechanisms to refine feature representations—such as channel and spatial attention, and multi-branch attention fusions—most apply fixed weighting or lack a data-driven mechanism to modulate attention adaptively based on the input context. Furthermore, few approaches explicitly incorporate mechanisms that can interpret the morphological characteristics of the input to drive the rebalancing of attention streams.

This research gap points to the need for a learnable, adaptive attention control strategy that not only captures rich semantic-spatial dependencies but also adjusts the fusion process dynamically to handle the heterogeneity of nodule shapes, sizes, and boundary uncertainty in real-world CT data.

#### 1.1.1. U-Net and Its Variants

U-Net (2015) [2]: Ronneberger et al. proposed U-Net, a symmetric encoder–decoder architecture featuring skip connections designed to effectively capture multi-scale features essential for medical image segmentation. However, its effectiveness diminishes when handling extremely small structures or noisy data.

U-Net++ (2019) [3]: Zhou et al. improved upon U-Net by introducing nested, densely connected skip connections to enhance multi-scale feature representation. Despite its improved accuracy, U-Net++ still faces challenges segmenting very small pulmonary nodules with unclear boundaries.

Res-UNet (2018) [4]: Zhang et al. incorporated residual blocks into the traditional U-Net architecture to alleviate gradient vanishing issues encountered during training. Although Res-UNet achieved notable results, it often struggled with accurately delineating small pulmonary nodules, particularly in noisy environments, resulting in Dice scores frequently below 85%.

#### 1.1.2. Attention-Based and Dual-Attention Mechanisms

To improve feature selection and localization, attention mechanisms have been incorporated into lung nodule segmentation models. While these methods enhance focus on relevant structures, many suffer from fixed attention weighting, which limits adaptability across different nodule morphologies.

Attention U-Net (2018) [5]: Oktay et al. proposed an extension of U-Net by integrating spatial attention, which allows the model to focus on salient regions., and they enhanced the standard U-Net by integrating attention gates, which selectively highlight relevant features while suppressing less important ones. This mechanism enables greater focus on salient regions, thereby improving segmentation accuracy, especially in medical imaging. By refining skip connections with attention, the model effectively captures fine details while maintaining contextual awareness.

DANet (2019) [6]: Fu et al. introduced the Dual-Attention Network (DANet), which simultaneously models channel and spatial attention to refine segmentation masks and thus enhance feature representation in segmentation tasks. By capturing long-range dependencies in both spatial and channel dimensions, it improves contextual understanding and feature refinement. This approach leads to more accurate segmentation, particularly in complex medical and natural image datasets.

MESAHA-Net (2023) [7]: This approach integrates multi-scale attention and hybrid attention mechanisms to enhance feature extraction and contextual learning. By combining spatial, channel, and hybrid attention, it effectively captures fine details while maintaining global dependencies. This design improves segmentation accuracy, particularly for high-resolution medical images, by balancing local and global feature representation.

#### 1.1.3. Transformer-Based Attention Models

Recent advancements in vision transformers have led to the development of Transformer-based attention models for lung nodule segmentation. These models excel at capturing global contextual dependencies but often require high computational resources and large-scale training datasets.

TransUNet (2021) [8]: Chen et al. introduced TransUNet, a hybrid Transformer-U-Net model that integrates self-attention mechanisms to capture long-range dependencies. However, its reliance on global self-attention increases computational costs, making it less feasible for real-time medical imaging applications.

MCAT-Net (2023) [9]: A multi-threshold and coordinate attention-based Transformer model that enhances both fine-grained feature extraction and global context awareness. Despite offering advantages, its computational complexity remains a significant drawback.

SW-UNet (2023) [10]: Ma et al. proposed SW-UNet, which integrates CNN with a sliding window Transformer to enhance segmentation performance. By leveraging local feature extraction from CNN and global attention modeling through a sliding window mechanism, the model captures multi-scale dependencies while reducing computational costs. However, its reliance on fixed window sizes may limit adaptability to varying lesion sizes and shapes.

### 1.2. Research Gaps

Despite significant advancements in deep-learning-based segmentation, lung nodule segmentation remains a challenging task due to boundary refinement issues, a lack of adaptive attention mechanisms, and computational inefficiency. Existing segmentation models suffer from inherent limitations that hinder their ability to achieve optimal performance across diverse nodule characteristics (see Table 1).

Beyond architectural limitations, another underexplored aspect lies in the choice of loss functions. Although widely used, region-based loss functions such as binary cross-entropy (BCE) and Dice loss are primarily designed to optimize global overlap, offering limited responsiveness to boundary-level discrepancies. This limitation becomes particularly significant in cases involving small or irregular nodules with subtle edge definitions. Consequently, incorporating boundary-aware losses—such as Sobel, Laplacian, and Hausdorff-based formulations—represents a promising direction to enhance edge sensitivity and support more precise segmentation in clinical contexts.

The remainder of this manuscript is structured as follows: Section 2 presents the materials and methods used to implement and train the proposed model. Section 3 provides results. Section 4 discusses the findings in the context of prior research. Finally, Section 5 concludes the paper and outlines future research directions.

## 2. Materials and Methods

### 2.1. Overall Architecture of CAAF-ResUnet

CAAF-ResUNet (Context-Aware Adaptive Attention Fusion ResUNet) is specifically developed for the accurate segmentation of lung nodules in computed tomography (CT) images, building upon the robust framework of Res-Unet++ [11]. This advanced model integrates two critical attention mechanisms—channel attention and position attention—complemented by an adaptive weighting mechanism designed to dynamically prioritize essential features based on the contextual nuances of each input image. The CAAF-UNet architecture comprises three primary components: encoder, bridge, and decoder.

The encoder is meticulously engineered for effective feature extraction, aiming to preserve critical spatial and textural information from the input CT images. It leverages residual convolutional blocks comprising 3 × 3 convolutions, batch normalization (BN), and rectified linear unit (ReLU) activations. This combination ensures training stability, mitigates information loss, and enables the capture of rich hierarchical representations. Residual connections incorporated throughout these blocks facilitate the direct flow of essential features across network layers, substantially reducing the degradation of information. Notably, the encoder adopts a hierarchical design, systematically extracting local details such as edges and textures at early layers (e.g., feature maps sized 64 × 64 × 32), and gradually progressing towards more abstract, global context features at deeper layers (feature maps sized 8 × 8 × 256). Such hierarchical processing ensures an optimal balance between detailed local representations and comprehensive contextual understanding, which is vital for precise segmentation.

A key innovation within the encoder structure is the Adaptive Attention Fusion (AAF) module. AAF distinctively adjusts the emphasis between channel attention, which prioritizes informative feature channels, and position attention, which highlights spatial dependencies [12]. This adaptive modulation is governed by the Adaptive Attention Controller (AAC), a novel component capable of dynamically generating optimal fusion weights tailored specifically to each input’s contextual characteristics. This mechanism effectively enables the network to adaptively emphasize the most informative features necessary for accurate segmentation. Detailed technical explanations of the AAC and its operational role within the AAF module are further elaborated in Section 2.3 and Section 2.4.

The bridge connects the encoder and decoder segments, playing a pivotal role in consolidating multi-scale feature representations. This segment employs the Atrous Spatial Pyramid Pooling (ASPP) module, utilizing multiple parallel convolutions with varied dilation rates (6, 12, 18). This strategy enables ASPP to effectively capture diverse spatial scales, from intricate local details to extensive global contexts. Furthermore, a global average pooling operation is included to encapsulate overall spatial context comprehensively. These multi-scale and global features are subsequently concatenated and refined through a 1 × 1 convolutional layer, maintaining dimensions consistent with the input (8 × 8 × 256). Consequently, ASPP significantly enriches global contextual representation, integrates multi-scale features efficiently, and preserves essential local details, thus enhancing the model’s capability to manage complex segmentation tasks accurately.

The decoder component is specifically designed to reconstruct precise segmentation maps by synthesizing and refining features originating from both the encoder and bridge modules. The decoder leverages skip connections strategically positioned at each resolution level, effectively transferring detailed spatial and textural information from the encoder directly to the corresponding decoder layers. Each decoder stage undertakes three core processes: first, it integrates encoder features with the features generated by preceding decoder layers; second, it enhances these combined features through an attention fusion mechanism; and third, it increases spatial resolution via upsampling layers.

Initially, the decoder fuses the bridge output (8 × 8 × 256) with the deepest encoder features (8 × 8 × 256), refining them through the AF module before upscaling to 16 × 16 × 128. Successive decoder stages repeat this integration, enhancement, and upsampling process, progressively generating finer-resolution feature maps of sizes 32 × 32 × 64 and 64 × 64 × 32. Ultimately, a concluding 1 × 1 convolutional layer is employed to reduce feature map channels to one, thereby producing the segmentation mask. The output map undergoes a Sigmoid normalization to ensure all pixel values are constrained within the [0, 1] range, facilitating the accurate delineation of lung nodules.

### 2.2. Network Pipeline and Processing Flow

**Figure 1:**
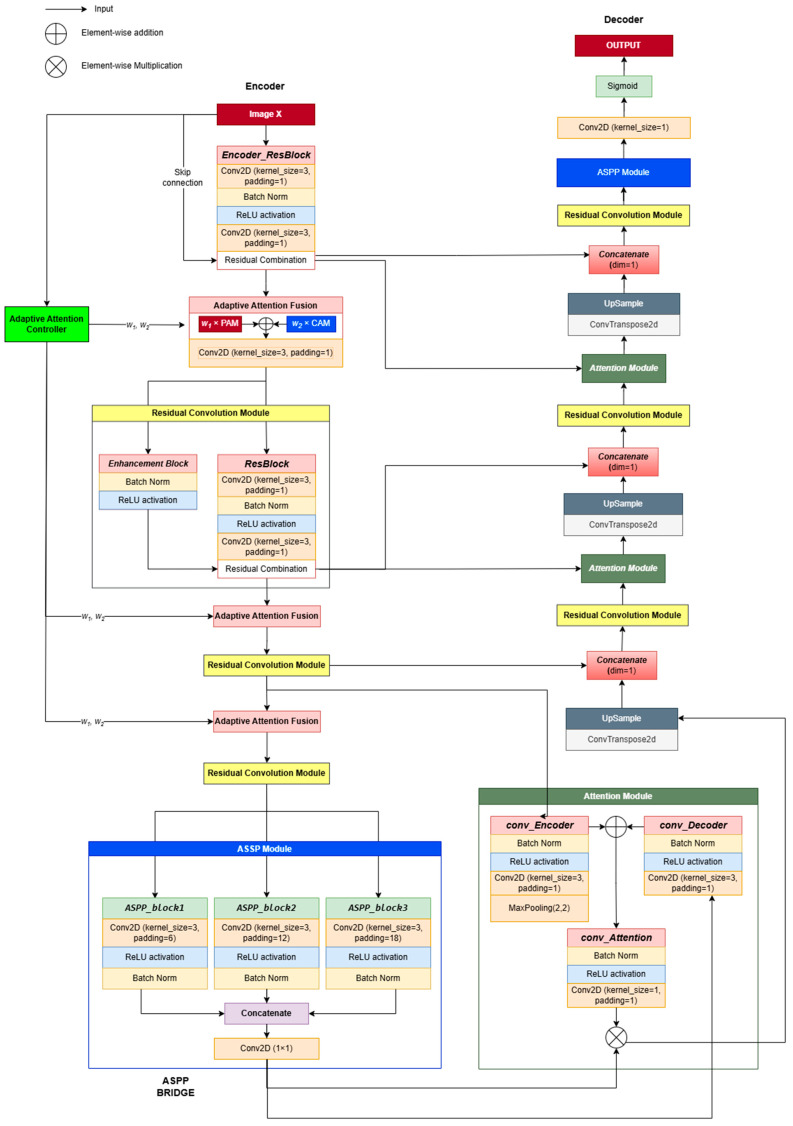
CAAF-Unet architecture. Each block is fully illustrated once and subsequently represented using the same color scheme to maintain consistency and clarity.

Given an input image X, the network processes it through multiple hierarchical stages (see in Figure 1):

Feature Encoding: The input X undergoes an initial convolutional transformation, producing an encoded feature representation F_0_. The Adaptive Attention Controller (AAC) processes a downsampled version of the input and generates adaptive attention weights (w_1_, w_2_) for attention fusion.

Adaptive Attention Fusion in the Encoder: Feature maps at each encoder stage are refined using Dual-attention Blocks, where AAF dynamically controls the balance between channel attention and position attention. The residual convolution operations ensure feature stability while downsampling.

Multi-Scale Representation in the Bottleneck: The lowest resolution feature maps pass through ASPP, which extracts multi-scale contextual features to enhance object boundary perception.

Attention-Guided Decoding: Encoder features are fused with decoder features through AAF-based upsampling blocks, progressively refining the segmentation map at each resolution level.

Final Output Generation: The last ASPP layer further enhances segmentation consistency before the sigmoid activation function generates the final probability mask.

### 2.3. Adaptive Attention Controller (AAC) with Self-Attention for Context-Aware Fusion

In encoder–decoder segmentation architectures, various attention mechanisms are commonly employed to enhance feature representations by emphasizing semantically important regions. However, integrating multiple attention branches (e.g., channel-wise and spatial attention) often results in static or manually designed fusion strategies, which may not generalize well across varying feature contexts. To address this limitation, we propose the Adaptive Attention Controller (AAC), a lightweight and versatile module capable of learning how to generate adaptive fusion weights based on the semantic characteristics of the input feature map. The detailed procedure of the AAC is illustrated in Algorithm 1.

The core idea of AAC is not to directly learn fixed fusion weights, but rather to learn how to dynamically produce adaptive weights conditioned on the contextual features of the input. This is achieved through a self-attention mechanism that identifies the interrelationships between spatial locations and generates a compact global representation, which is then used to derive fusion weights in a flexible and input-dependent manner.
**Algorithm 1:** Adaptive Attention Controller using Self-Attention.**Input:** $\;\mathrm{Feature}\;\mathrm{map}\;F_{in}\in R^{B\times C\times H\times W}$, downsample size *H*′ × *W*′ (default: 8 × 8)$\mathrm{Output}:\;\mathrm{Adaptive}\;\mathrm{weights}\;w\in R^{B\times 2}$**Step 1:** Downsample the input feature map via adaptive average pooling.$F_{ds}=AdaptiveAvgPool\left(F_{in},\left(H^{\prime },W^{\prime }\right)\right)\in R^{B\times C\times H^{\prime }\times W^{\prime }}.$(1)**Step 2:** Reshape the downsampled features into spatial sequences.$F_{seq}=Reshape\left(F_{ds}\right)\in R^{B\times N\times C},N=H^{\prime }\times W^{\prime }.$(2)**Step 3:** Project spatial sequences into query (*Q*), key (*K*), and value (*V*) vectors.$\begin{array}{c} Q=F_{seq}W_{Q},\;K=F_{seq}W_{K},V=F_{seq}W_{V}\\ W_{Q},W_{K},W_{V}\in R^{C\times C}. \end{array}$,(3)**Step 4:** Compute self-attention maps via scaled dot-product attention.$A=Softmax\left(\frac{QK^{⊤}}{\sqrt{C}}\right)\in R^{B\times N\times N}.$(4)**Step 5:** Compute the attention-refined feature vectors.$F_{sa}=AV\in R^{B\times N\times C}.$(5)**Step 6:** Extract global feature representation via spatial averaging.$F_{\mathrm{global}\;}=\frac{1}{N}\sum_{i=1}^{N}\;F_{sa}^{\left(i\right)}\in R^{B\times C}.$(6)**Step 7:** Project global features into intermediate representation using a fully connected layer with *ReLU* activation.$h=ReLU\left(F_{\mathrm{global}\;}W_{1}+b_{1}\right)\in R^{B\times \frac{C}{2}},W_{1}\in R^{C\times \frac{C}{2}}.$(7)**Step 8:** Compute output scores using another fully connected layer.$z=hW_{2}+b_{2}\in R^{B\times 2},W_{2}\in R^{\frac{C}{2}\times 2}.$(8)**Step 9:** Convert scores into adaptive weights via softmax normalization.$w=Softmax\left(z\right)\in R^{B\times 2}.$(9)**Step 10:** Return adaptive weights w.

To better understand the functioning of AAC, we decompose the algorithm into three principal steps as outlined below:

(A) Spatial Context Encoding: Steps 1–2 map the high-resolution feature map to a reduced spatial domain. This downsampling not only helps decrease computational costs but also enables the AAC to concentrate on the overall semantic layout rather than local details. The resulting representation is reshaped into a spatial sequence, facilitating attention-based modeling across spatial positions.
(B) Self-Attention Mechanism: Steps 3–5 apply self-attention to a sequence of spatial feature vectors. Specifically, from the input feature map of size *C* × *H*′ × *W*′, each spatial location is represented by a *C*-dimensional feature vector. These spatial vectors are then flattened in a consistent order (e.g., row-wise) to form a sequence of length *N* = *H*′ × *W*′, enabling the application of attention across spatial positions as in Transformer-based models. For each position in the sequence, a query vector is generated and compared with key vectors from all other positions to compute a softmax-normalized attention map that determines the relative importance of each spatial location. Weighted summation of value vectors using the attention map results in an updated representation at each position. As a result, each spatial vector is contextually enriched with information from all other positions, enabling the model to capture long-range dependencies. A notable contribution of AAC is its use of reduced spatial resolution (e.g., 8 × 8) before applying self-attention. This significantly reduces the computational cost from *O* ((*H* × *W*)^2^) *to O*(64^2^) while preserving the essential global semantic structure. The reduction acts as a semantic filter, focusing the model’s attention on broader structures rather than noisy local details.
(C) Adaptive Weight Generation: Steps 6–8 aggregate the attention-enhanced features into a compact global representation. Through a two-layer fully connected network with ReLU activation and softmax normalization, the AAC computes the final adaptive weights, which can be directly used to fuse the outputs from different attention branches.

The computational complexity of the AAC module is dominated primarily by the self-attention calculation. The complexity analysis of each significant step is summarized in Table 2.

Considering *N* = *H*′ × *W*′, the total computational complexity of AAC can be expressed as

*O* (*B* × *N*^2^ × *C*) = *O* (*B* × *H*′^2^ × *W*′^2^ × C) (10)

Our choice of *H*′ = *W*′ = 8 ensures that AAC maintains a high representational capacity while significantly controlling computational complexity, making it suitable for practical segmentation tasks.

### 2.4. Adaptive Attention Fusion (AAF)

Traditional attention fusion methods often apply fixed or manually determined weight distributions, which may not be optimal for complex medical image segmentation tasks. These approaches fail to adapt to the varying significance of spatial and channel-wise features, leading to suboptimal feature representation. To address this, AAF introduces a dynamic weighting mechanism, allowing the model to intelligently adjust the influence of channel attention (CA) and position attention (PA) at different feature levels. Instead of assigning static importance to spatial and channel features, AAF leverages fusion coefficients (w_1_,w_2_) generated by the AAC to regulate feature integration in a data-driven manner. This adaptive approach ensures that spatial structures and channel-specific information contribute proportionally to feature refinement, depending on the characteristics of the input. By guiding attention fusion based on learned contextual relevance, AAF improves discriminative feature selection while minimizing redundant activations, making it particularly well-suited for boundary-sensitive medical image segmentation.

Mechanism of AAF: Optimizing Feature Integration

AAF processes the feature maps refined by channel attention (CA) and position attention (PA) and adaptively modulates their contributions using the fusion coefficients w_1_ and w_2_, which are derived from AAC. Instead of treating spatial and channel attention equally, AAF assigns context-aware importance to each component, ensuring that informative features are selectively enhanced while irrelevant details are suppressed. This strategy enables the model to achieve a more effective feature integration process, leading to improved segmentation accuracy.

Let the input feature map be designated as *X*; the outputs of channel attention and position attention are represented as follows:

X_CA_ = CA(X), X_PA_ = PA(X). (11)

The final fused feature representation is computed as follows:

(12) $$X_{AAF}=w_{1}X_{PA}+w_{2}X_{CA}.$$

The core advantage of AAF lies in its adaptive nature, which contrasts with traditional fusion methods that often apply rigid weighting schemes. This adaptability provides several key benefits, as shown in Table 3.

### 2.5. Boundary-Aware Loss Function (BAL)

#### 2.5.1. Formulation of Boundary-Aware Loss

Segmentation models, particularly in medical imaging, require not only accurate region classification but also precise boundary delineation to distinguish anatomical structures effectively. Traditional loss functions such as Dice loss and cross-entropy loss primarily focus on pixel-wise classification accuracy but often struggle with boundary ambiguity, leading to imprecise segmentation edges. To address this challenge, we introduce a boundary-aware loss (BAL) that combines Dice loss with a boundary-sensitive component derived from Sobel and Laplacian edge detection operators. The proposed loss function enhances spatial awareness by incorporating boundary constraints, ensuring that segmentation predictions not only capture the target region but also align closely with its true contours. By penalizing discrepancies along object boundaries, BAL improves fine-detail segmentation, reducing errors in cases where adjacent structures exhibit similar intensity distributions.

Dice loss ($L_{Dice}$) is a widely used metric for segmentation tasks, designed to optimize the overlap between the predicted mask *P* and the ground truth *G*. It is formulated as follows:

(13) $$L_{\mathrm{Dice}\;}=1-\frac{2\sum PG}{\sum P+\sum G}.$$

To further refine segmentation along object contours, the boundary loss ($L_{Boundary}$) penalizes discrepancies between the predicted and actual boundaries. Unlike Dice loss, which primarily optimizes volumetric overlap, boundary loss explicitly focuses on edge precision. In subsequent sections, we introduce three computational strategies for deriving $L_{Boundary}$:

- Sobel filtering, which enhances edge structures by detecting intensity variations.
- Laplacian operators, which emphasize second-order spatial derivatives to refine boundary sharpness.
- Hausdorff distance, which quantifies the worst-case discrepancy between predicted and ground truth contours.

The boundary-aware loss (*BAL*) is formulated as follows:

(14) $$L_{BAL}=L_{\mathrm{Dice}\;}+L_{\mathrm{Boundary}\;}.$$

#### 2.5.2. Sobel-Based Boundary Loss

In segmentation tasks, one of the primary challenges is ensuring that the predicted boundary of an object closely aligns with the ground truth. A misaligned boundary can lead to over-segmentation (including irrelevant regions) or under-segmentation (missing parts of the target), both of which negatively impact the model’s reliability. To formally define the boundary discrepancy, we consider the predicted segmentation mask *P* and the ground truth mask *G*. The boundary regions of these masks, denoted as *∂P* and *∂G*, represent the set of pixels where the intensity gradient is high, indicating significant structural transitions. The boundary loss, in its ideal form, is computed as the pixel-wise difference between these extracted boundary maps:

(15) $$L_{\mathrm{Boundary}\;}=\sum_{i,j}\;\left|\partial P_{i,j}-\partial G_{i,j}\right|.$$

where *i,j* represent spatial coordinates, and the absolute difference ensures that misaligned boundaries are explicitly penalized. However, directly extracting *∂P* and *∂G* from segmentation masks is non-trivial, as these masks are often binary and lack smooth intensity variations. To overcome this, we employ the Sobel operator, a widely used method for computing image gradients, to approximate object boundaries efficiently.

The Sobel operator is a discrete convolutional filter that estimates the first-order derivatives of an image, making it effective for detecting intensity transitions along object boundaries. It consists of two directional filters:

The horizontal gradient (*Gx*) is computed using the convolution kernel:

(16) $$S_{x}=\left[\begin{array}{ccc} -1 & 0 & 1\\ -2 & 0 & 2\\ -1 & 0 & 1 \end{array}\right].$$

The vertical gradient (*G_y_*) is computed using the kernel:

(17) $$S_{y}=\left[\begin{array}{ccc} -1 & -2 & -1\\ 0 & 0 & 0\\ 1 & 2 & 1 \end{array}\right]$$

Applying these filters to the predicted segmentation mask *P* and the ground truth *G*, we obtain their respective gradient magnitudes, which serve as boundary approximations:

(18) $$\partial P=\sqrt{{\left(P*S_{x}\right)}^{2}+{\left(P*S_{y}\right)}^{2}};\;\partial G=\sqrt{{\left(G*S_{x}\right)}^{2}+{\left(G*S_{y}\right)}^{2}}.$$

where ∗ denotes the convolution operation. The Sobel-based boundary loss is then formulated as follows:

(19) $$L_{\mathrm{Boundary}\;}=\sum_{i,j}\;\left|\sqrt{{\left(P*S_{x}\right)}^{2}+{\left(P*S_{y}\right)}^{2}}-\sqrt{{\left(G*S_{x}\right)}^{2}+{\left(G*S_{y}\right)}^{2}}\right|.$$

By computing the L1 norm difference between the predicted and actual boundary gradients, this loss function ensures that the segmentation network learns to produce contours that closely align with ground truth edges. The detailed computation of the loss function is presented in Algorithm 2.
**Algorithm 2:** Sobel-Based Boundary Loss ComputationGiven predicted and ground truth segmentation masks *P* and *G*, the Sobel loss computation follows these steps:**Step 1.** Compute Gradient Magnitude.Apply the Sobel operator to *P*, obtaining *M_P_*.Apply the Sobel operator to *G*, obtaining *M_G_*.**Step 2.** Compute Edge Discrepancy.Compute the absolute difference between *M_P_* and *M_G_*:$D=\left|M_{P}-M_{G}\right|$(20)**Step 3.** Aggregate the Loss.Compute the final Sobel loss as the mean or sum of the absolute differences:$L_{\mathrm{sobel}\;}=\frac{1}{N}\sum_{i,j}\;D(i,j)$(21)where N is the total number of pixels.

#### 2.5.3. Laplacian-Based Loss Function

The Laplacian operator is a second-order differential operator that quantifies the rate of change in the gradient. It is mathematically defined as follows:

(22) $$\nabla^{2}f=\frac{\partial^{2}f}{\partial x^{2}}+\frac{\partial^{2}f}{\partial y^{2}}$$

This operator characterizes local variations in a function by summing second-order partial derivatives along all spatial dimensions. In image analysis, the Laplacian highlights regions of rapid intensity change, making it particularly effective in detecting edges and structural variations. From a differential geometry perspective, the Laplacian describes how much a function deviates from its local mean, allowing it to serve as a boundary-sensitive operator. It is particularly useful in cases where object edges must be accurately captured, as it enhances high-frequency components while suppressing uniform regions. By leveraging the Laplacian’s sensitivity to curvature and shape irregularities, segmentation models can benefit from enforcing structural consistency in the predicted mask relative to the ground truth. This approach ensures that the model learns to minimize not just pixel-wise discrepancies but also shape deformations, preserving anatomical structures in medical imaging applications.

To transition from continuous mathematics to computational implementation, we approximate the Laplacian operator using discrete convolution kernels. The most common discrete approximation in a 2D Cartesian grid is defined as follows:

(23) $$\nabla^{2}I\approx I(x+1,y)+I(x-1,y)+I(x,y+1)+I(x,y-1)-4I(x,y)$$

which can be represented as a convolution with the following kernel:

(24) $$\left[\begin{array}{ccc} 0 & 1 & 0\\ 1 & -4 & 1\\ 0 & 1 & 0 \end{array}\right]$$

where each pixel *I(x,y)* is replaced by the weighted sum of its neighboring pixels. This discrete form serves as a computationally efficient method for approximating second-order derivatives, allowing segmentation models to process image structures effectively.

Applying this discrete Laplacian to both the predicted mask *P* and the ground truth *G* enables the computation of boundary-aware differences. The absolute difference between the Laplacian-transformed outputs serves as a metric for structural deviation, reinforcing segmentation models with spatially coherent predictions.

This formulation bridges the gap between mathematical foundations and machine learning applications, allowing segmentation models to integrate geometric priors into their loss functions for improved boundary precision.

To incorporate the Laplacian operator into a loss function for segmentation, we define the Laplacian loss as the absolute difference between the second-order derivatives of the predicted mask *P* and the ground truth mask *G*:

(25) $$L_{lap}=\sum_{i,j}\;\left|\nabla^{2}P_{i,j}-\nabla^{2}G_{i,j}\right|$$

where $\Delta^{2}P\;$and $\Delta^{2}G\;$are the Laplacian-transformed versions of the predicted and ground truth masks, respectively, and *i,j* represent index pixel coordinates. The key steps of the boundary-aware loss computation are summarized in Algorithm 3.
**Algorithm 3:** Laplacian-Based Boundary Loss Computation**Step 1**. Compute Laplacian Transformations.Apply the discrete Laplacian operator to the predicted mask *P*, obtaining$L_{P}=\nabla^{2}P$(26)Apply the same operator to the ground truth mask *G*, obtaining *L_G_***Step 2.** Calculate the Boundary Discrepancy.Compute the absolute difference between LP and LG at each pixel location:$D=\left|L_{P}-L_{G}\right|$(27)**Step 3.** Aggregate the Loss.Compute the final Laplacian loss as the mean or sum of the absolute differences:$L_{lap}=\frac{1}{N}\sum_{i,j}\;D_{i,j}$(28)where *N* is the total number of pixels.

#### 2.5.4. Hausdorff-Based Loss Function

Accurate medical image segmentation requires both regional overlap and precise boundary alignment. While Dice loss captures volumetric agreement, it often overlooks fine boundary details. To address this, we incorporate Hausdorff distance loss, which penalizes the largest spatial deviations between predicted and ground-truth boundaries, thus enhancing contour accuracy. A brief description is given in Algorithm 4.

(29) $$\begin{array}{rr} & H(P,G)=max\left(\underset{p\in P}{sup}\;\underset{g\in G}{inf}\;d(p,g),\underset{g\in G}{sup}\;\underset{p\in P}{inf}\;d(g,p)\right), \end{array}$$

where *d(p,g)* is the Euclidean distance between points *p* and *g*. Since direct computation is inefficient, we approximate it using a Sobel-based edge extraction method. To approximate the Hausdorff distance, we first extract edge maps from the segmentation masks using the Sobel operator. The Sobel filters for horizontal and vertical gradient estimation are given by

(30) $$G_{x}=\left[\begin{array}{lll} 1 & 0 & -1\\ 2 & 0 & -2\\ 1 & 0 & -1 \end{array}\right],G_{y}=\left[\begin{array}{ccc} 1 & 2 & 1\\ 0 & 0 & 0\\ -1 & -2 & -1 \end{array}\right].$$

The edge gradients in the horizontal and vertical directions are first computed using convolution:

(31) $$I_{x}=G_{x}*I,I_{y}=G_{y}*I$$

The edge magnitude is then calculated as follows:

(32) $$M=\sqrt{I_{x}^{2}+I_{y}^{2}}$$

By extracting edges from both prediction and ground truth masks, we compute the directed Hausdorff distance between boundary points.
**Algorithm 4:** Hausdorff-Based Boundary Loss Computation  Given predicted and ground truth segmentation masks *P* and *G*, the loss computation follows these steps:**Step 1:** Extract Edge Maps.Apply Sobel filters to *P* and *G* to obtain *E_P_* and *E_G_*.**Step 2:** Compute Directed Hausdorff Distances.For each edge pixel in *E_P_*, find the nearest edge pixel in *E_G_*.For each edge pixel in *E_G_*, find the nearest edge pixel in *E_P_*.Compute mean minimum distances in both directions.

While Sobel-based edge extraction provides a simple and interpretable way to approximate Hausdorff distance, it suffers from key limitations: the computation is non-differentiable, sensitive to minor pixel shifts, and computationally expensive due to pairwise nearest-neighbor comparisons between edge pixels, resulting in a time complexity of O(n × m). To overcome these issues, we adopt an alternative formulation using the Euclidean Distance Transform (EDT). Instead of relying on explicit edge matching, EDT produces continuous-valued distance maps, enabling efficient and fully differentiable approximation of directed Hausdorff distances. This approach is not only compatible with gradient-based optimization but also significantly reduces computational cost via linear-time operations. The procedure for computing the boundary-guided loss function is detailed in Algorithm 5.

This approach does not require explicit edge detection and instead computes continuous distance maps directly from binary segmentation masks. Given a predicted mask $P\in \{0,1{\}}^{H\times W}$ and a ground truth mask $G\in \{0,1{\}}^{H\times W}$, we define the following:

$D_{G}=EDT(1-G)$: a distance map representing the distance from each background pixel to the nearest foreground pixel in *G*.

$D_{P}=EDT(1-P):\;$similarly, the distance from each background pixel to the nearest foreground pixel in *P*.

The directed distances from *P* to *G* and from *G* to *P* are computed as follows:

(33) $$d(P\to G)=\frac{1}{\left|P\right|}\sum_{p\in P}\;D_{G}(p)\;\mathrm{and}\;d(G\to P)=\frac{1}{\left|G\right|}\sum_{g\in G}\;D_{P}(g)$$

where *P* and *G* denote the sets of foreground pixels in the predicted and ground truth masks, respectively. $D_{G}(p)$ is the EDT value from the pixel p∈P to the closest pixel in *G*, and vice versa. The final symmetric EDT-based Hausdorff loss is defined as follows:

(34) $$L_{\mathrm{EDT}\text{-}\mathrm{Hausdorff}\;}=d(P\to G)+d(G\to P)$$

**Algorithm 5:** Hausdorff Loss Computation using EDT**Input:**$\;\mathrm{Binary}\;\mathrm{predicted}\;\mathrm{mask}\;P\in \{0,1{\}}^{H\times W}$$\mathrm{Binary}\;\mathrm{ground}\;\mathrm{truth}\;\mathrm{mask}\;G\in \{0,1{\}}^{H\times W}$**Output:**$\;\mathrm{Hausdorff}\text{-}\mathrm{based}\;\mathrm{loss}\;\mathrm{value}\;L_{\mathrm{EDT}\text{-}\mathrm{Hausdorff}\;}$**Step 1:** Compute distance transform from ground truth.$D_{G}=EDT(1-G)$(35)**Step 2:** Compute distance transform from prediction.$D_{P}=EDT(1-P)$(36)**Step 3:** Compute directed distances.$\begin{array}{ll} d_{PG}=mean\left(D_{G}[p]\right), & \forall p\in P\\ d_{GP}=mean\left(D_{P}[g]\right), & \forall g\in G \end{array}$(37)**Step 4:** Compute final loss.$L_{\mathrm{EDT}\text{-}\mathrm{Hausdorff}\;}=d_{PG}+d_{GP}$(38)

### 2.6. Experimental Setup

#### 2.6.1. Evaluation Metrics

Evaluating segmentation models requires suitable and significant measures to fairly represent performance. We use the Dice score, Intersection over Union (*IoU*), sensitivity, specificity, and Miss Rate measures in the segmentation problem on CT lung nodules to provide a whole picture of the model’s strengths. The metrics are derived from the values of the confusion matrix: true positive (*TP*), true negative (*TN*), false positive (*FP*), and false negative (*FN*) values. *TP* denotes the accurately segmented pixels of pulmonary nodules; *TN* denotes the pixels accurately classified as non-nodules; *FP* represents the pixels misclassified as nodules, signifying over-segmentation; and *FN* represents the quantity of nodule pixels overlooked by the model, indicating under-segmentation.

The Dice score is favored as it immediately assesses segmentation quality by evaluating the extent of the overlap.

(39) $$Dice=\frac{2\times TP}{\left(2\times TP+FN+FP\right)}$$

Intersection over Union (*IoU*) is a typical metric used to assess the overlap between the predicted segmentation area and the actual area. In the *IoU* formula, the weight for *TP* is not doubled, making this metric balanced between *FP* and *FN*, making it more suitable for evaluating overall accuracy in problems with residual errors (*FP*) or omission errors (*FN*).

(40) $$IoU=\frac{TP}{\left(TP+FN+FP\right)}$$

Sensitivity, referred to as the true positive rate (TPR) or recall, quantifies the model’s capacity to accurately identify true positive (*TP*) values. In the realm of lung nodule image segmentation, this metric indicates the model’s efficacy in identifying real lung nodules.

(41) $$Sensitivity=\frac{TP}{\left(TP+FN\right)}$$

The Miss Rate, often referred to as the false negative rate (FNR), indicates the percentage of lung nodules that the model fails to identify. A high Miss Rate indicates that the model fails to detect numerous lung nodules, which poses significant risks in medicine, since overlooked lung nodules may result in delayed or erroneous diagnoses.

(42) $$Miss\;Rate=\frac{FN}{\left(TP+FN\right)}$$

Specificity denotes the ratio of non-lung nodule regions (true negatives) accurately recognized by the model, guaranteeing that no false positives occur when normal regions in the image are misidentified as abnormalities. This is significant in healthcare since false positives can result in superfluous testing and induce unwarranted concern for patients.

(43) $$Specificity=\frac{TN}{\left(TN+FP\right)}$$

#### 2.6.2. Datasets

To evaluate the effectiveness of our proposed method, we conducted experiments on three datasets: LIDC-IDRI (Lung Image Database Consortium and Image Database Resource Initiative) [13]; LUNA16 (Lung Nodule Analysis 2016) [14]; and an in-house clinical dataset (MEF-PN35) collected from the Department of Diagnostic Imaging at the University Medical Center Ho Chi Minh City, Vietnam. These datasets provide a comprehensive benchmark for developing and assessing deep learning models in lung nodule segmentation. The dataset characteristics are summarized in Table 4.

#### 2.6.3. Experimental Design

In this section, we describe the detailed experimental procedures conducted to comprehensively evaluate the effectiveness and robustness of our proposed CAAF-ResUNet model for lung nodule segmentation. The segmentation models were implemented using the PyTorch version 1.8.0 framework and trained on an NVIDIA A100 GPU via Google Colab Pro+. Training was conducted using the AdamW optimizer with an initial learning rate of 0.0001, ensuring better weight decay handling and preventing the learning rate from decaying to zero. A StepLR scheduler was applied, reducing the learning rate by a factor of 0.1 every 30 epochs to stabilize training.

Experiment 1: Comparison of Boundary-Aware Loss Functions

This experiment compares three boundary-aware loss configurations—Dice score combined with Sobel, Laplacian, or Hausdorff loss—to assess their impact on segmentation accuracy. Evaluated on the LUNA16 and LIDC-IDRI datasets using the Dice score, IoU, sensitivity, specificity, and Miss Rate, the results show that Hausdorff-based loss offers superior boundary adherence and effectively reduces false negatives.

Experiment 2: Effectiveness of the Adaptive Attention Controller (AAC)

This experiment investigates the impact of the Adaptive Attention Controller (AAC) on segmentation performance by comparing two model configurations: one employing AAC to dynamically modulate attention weights, and the other using fixed attention weights set to w_1_ = w_2_ = 0.5. This comparison aims to isolate the effectiveness of adaptive versus static attention mechanisms in feature fusion.

Both models utilize the same encoder–decoder backbone. The key difference lies in the attention fusion mechanism: adaptive vs. static. The evaluation, conducted on the LIDC-IDRI dataset, shows that the AAC leads to consistent performance gains, particularly in the Dice score and Miss Rate, by effectively adapting attention contributions to nodule morphology.

Experiment 3: Clinical Validation on Real-World CT Data

To assess clinical applicability, the model was validated on MEF-PN35—a real-world dataset comprising 804 CT slices from 35 patients with diverse nodule morphologies. Performance was evaluated using the Dice score, sensitivity, specificity, and Miss Rate. Additionally, Grad-CAM visualizations were employed to interpret attention consistency, offering qualitative insights into the model’s focus on lesion regions in realistic clinical scenarios.

## 3. Results

This section presents a comprehensive evaluation of the proposed CAAF-ResUNet model through three main experiments: (1) comparing boundary-aware loss functions (Sobel, Laplacian, Hausdorff) combined with Dice loss, (2) analyzing the effect of the Adaptive Attention Controller (AAC) and its learned fusion weights (w_1_, w_2_), and (3) validating the model’s generalization on both public and clinical CT datasets. The experiments assess segmentation performance using the Dice coefficient, Intersection over Union, sensitivity, specificity, and Miss Rate, all reported as mean ± standard deviation.

### 3.1. Comparison of Boundary-Aware Loss Functions in the CAAF-ResUNet Model

We investigated the impact of boundary-aware loss design by comparing three configurations—Dice combined with Sobel, Laplacian, and Hausdorff operators—within the CAAF-ResUNet framework. The model demonstrates strong segmentation performance, primarily due to its adaptive attention architecture. The Adaptive Attention Controller (AAC) dynamically adjusts the contributions of channel and position attention based on input morphology, while the Adaptive Attention Fusion (AAF) aggregates spatial features to enhance boundary precision. This integration enables the model to maintain morphological consistency even in nodules with ambiguous or complex contours.

Experiments were performed on two publicly accessible datasets, LUNA16 and LIDC-IDRI, to assess the generalization of each loss function under varying imaging conditions and nodule attributes. This comparison aimed to evaluate the balance between segmentation accuracy and stability, as well as to determine the best-performing and clinically applicable loss configuration for incorporation into the final model.

**Figure 2:**
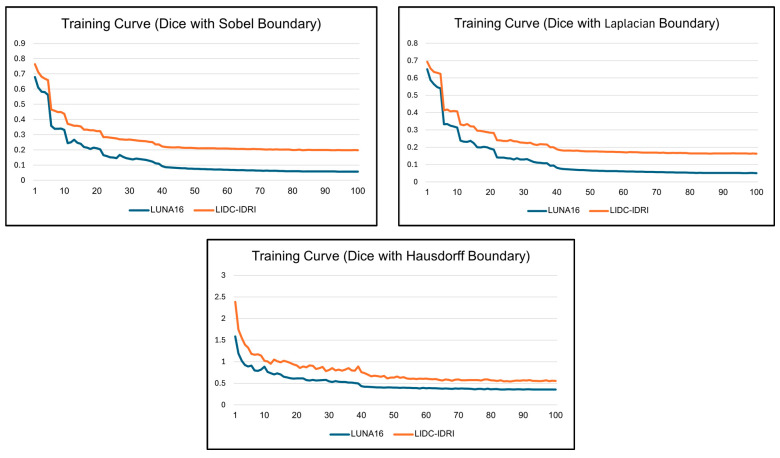
Training loss curves of CAAF-ResUNet using boundary-aware loss functions, each comprising Dice loss combined with either Sobel, Laplacian, or Hausdorff terms. Results are shown on LUNA16 and LIDC-IDRI over 100 epochs. Sobel-based loss converged rapidly and smoothly, Laplacian showed early fluctuations, while Hausdorff maintained higher values due to its global alignment nature.

Figure 2 depicts the training loss trajectories throughout 100 epochs for each configuration. The Sobel-based configuration showed the highest efficiency and stability in convergence behavior. Beginning with early loss values of roughly 0.76 on LIDC-IDRI and 0.68 on LUNA16, the losses diminished swiftly and settled below 0.3 by epoch 50, ending in final values of around 0.19 and 0.05, respectively. The Laplacian-based design showed stronger oscillations during the first training period, especially on LIDC-IDRI, although starting from comparable beginning values (~0.70). The loss progressively stabilized post-epoch 30, converging to around 0.16 for LIDC-IDRI and 0.05 for LUNA16 at the conclusion of training. On the other hand, the Hausdorff-based configuration, executed through the Euclidean Distance Transform (EDT), initiated with significantly elevated loss values—around 2.4 on LIDC-IDRI and 1.6 on LUNA16—and maintained a consistent decline during training, finally stabilizing at approximately 0.55 and 0.35, respectively, by epoch 100.

Table 5 encapsulates the quantitative segmentation efficacy for each loss setting across the two datasets. In the LUNA16 dataset, the Hausdorff-based configuration attained the highest Dice score (90.88 ± 6.16)% and the lowest Miss Rate (8.92 ± 10.20)%, with the Sobel-based configuration closely following. The Laplacian-based configuration demonstrated reduced Dice and IoU scores, accompanied by increased volatility. Comparable trends were noted in the LIDC-IDRI dataset. The Hausdorff arrangement produced the highest Dice coefficient (85.92 ± 16.70)% and sensitivity (92.38 ± 16.88)%, along with the lowest Miss Rate (7.62 ± 16.88)%. The Sobel-based loss showed similar performance; however, the Laplacian setup produced less reliable segmentation quality.

The values of w_1_ and w_2_ presented in Table 5 denote the mean dynamic weights over all test samples, as generated by the AAC. The weights are dynamic characteristics derived on a per-sample basis, contingent upon the morphological context of each input. On both LUNA16 and LIDC-IDRI, the controller consistently prioritized channel attention (w_2_) over position attention (w_1_), especially under the Hausdorff configuration (e.g., mean w_2_ = 0.74 and w_1_ = 0.26 on LUNA16; mean w_2_ = 0.78 and w_1_ = 0.22 on LIDC-IDRI).

### 3.2. Effectiveness of the Adaptive Attention Controller (AAC) Module

To comprehensively evaluate the effectiveness of the Adaptive Attention Controller (AAC), we first conducted an internal comparison within the CAAF-ResUNet framework. In this experiment, two variants of the model were trained—one with AAC and one without—while keeping all other architectural components and training protocols constant. This controlled setup allowed us to isolate and quantify the specific contribution of AAC under three boundary-aware loss configurations, where the standard Dice loss was combined with edge-sensitive strategies including Laplacian filtering, Sobel operators, and Hausdorff distance. As shown in Table 6, the incorporation of AAC consistently improved segmentation performance across all tested settings. To validate the statistical significance of these improvements, we conducted paired *t*-tests on the Dice scores produced by the AAC and non-AAC variants on the same 1200 test cases from the LUNA16 dataset.

The *p*-value was computed based on the differences between these paired results. Let *d*_*i*_ = *x*_*i*_ – *y*_*i*_ denote the difference in Dice scores between the AAC-enabled model *x*_*i*_ and the non-AAC model *y*_*i*_ for the *i*-th image. Given these 1200 paired differences, the *p*-value was calculated using a two-tailed paired *t*-test, which estimates the probability that the observed mean difference could occur by random chance under the null hypothesis of no true difference.

Table 6 summarizes the statistical significance of performance differences between AAC and non-AAC model variants under various boundary-aware loss configurations. The results were obtained using paired *t*-tests on Dice scores computed from the same set of test cases. Across all settings, the inclusion of AAC led to statistically significant improvements, as evidenced by consistently low *p*-values. These findings confirm that the Adaptive Attention Controller provides a consistent and meaningful enhancement in segmentation accuracy, regardless of the specific loss function applied.

To further assess the impact of the Adaptive Attention Controller (AAC), we evaluated additional model configurations with and without AAC integration, as presented in Table 7.

In the LUNA16 dataset, the comprehensive model CAAF-ResUNet (utilizing AAC and Hausdorff loss) attained the superior Dice score of 90.88%, surpassing static attention configurations including Position + ResUNet (82.79%), Channel + ResUNet (84.44%), PCAM + ResUNet with fixed weights (85.96%), and the baseline ResUNet++ (83.89%).

The comparison on the more intricate LIDC-IDRI dataset was confined to CAAF-ResUNet and PCAM + ResUNet. The AAC-enabled device achieved a Dice score of 85.92%, whilst the fixed-weight setup recorded 85.37%. The AAC module generated asymmetric attention weights across both datasets, yielding average values of w_1_ = 0.22 and w_2_ = 0.78 under Hausdorff supervision.

### 3.3. Comparative Analysis with Existing Models

Table 8 provides a comparative evaluation of the lung nodule segmentation performance between CAAF-ResUNet and several prominent deep learning models using the LUNA16 and LIDC-IDRI datasets. CAAF-ResUNet demonstrated Dice similarity coefficients of 90.88% and 85.92% on LUNA16 and LIDC-IDRI, respectively, outperforming several other state-of-the-art models.

The U-Det and Bidirectional Feature Network previously showed robust performance on the LUNA16 dataset, both attaining a DSC of 82.82%. Nonetheless, these structures are deficient in adaptive attention fusion processes, constraining their capacity to effectively address local and global contextual fluctuations. The Dual Encoding Fusion Network attained the highest performance on the LIDC-IDRI dataset, with a DSC of 85.27%, surpassing previous methodologies. This approach employs a sophisticated multi-branch feature integration method but does not utilize dynamic attention balancing.

Recently, SCA-VNet [15] has demonstrated promising results (Dice = 87.50%) on the LIDC-IDRI dataset by combining 3D spatial and channel attention mechanisms with residual edge-enhancement modules. This approach benefits from volumetric context and offers robust segmentation in 3D settings. In contrast, CAAF-ResUNet follows a 2D slice-based approach with adaptive attention fusion and boundary-aware supervision. While it does not exploit 3D spatial continuity, it is more lightweight and adaptable to real-world clinical environments where full 3D annotations may not always be available. This design makes it more practical in scenarios with limited data or computational constraints.

Conversely, CAAF-ResUNet implements an AAF technique that integrates channel and positional attention, dynamically regulated by the AAC. This design allows the model to address diverse anatomical and border issues, enhancing segmentation accuracy—especially in noisy or structurally confusing areas.

**Table 8:** Comparative performance of CAAF-ResUNet and existing lung nodule segmentation models on the LUNA16 and LIDC-IDRI datasets. Dice score (%) is reported to highlight improvements achieved through adaptive attention fusion.

Authors	Model	Dice Score (%)
Tong et al. (2018) [16]	Unet	82.05
Keetha et al. (2020) [17]	U-Det	82.82
Wu et al. (2021) [18]	Dual-branch network	83.16
Chen et al. (2021) [19]	Fast multi-crop guided attention network	81.32
Xu et al. (2022) [20]	Dual encoding fusion network	85.27
Maqsood et al. (2021) [21]	DA-Net	81.00
Sekhara et al. (2023) [22]	Bidirectional feature network	82.82
Liu et al. (2024) [15]	SCA-Vnet	87.50
Our model	ResUnet + AAF + Boundary-aware loss	90.88

### 3.4. Clinical Validation on Real-World CT Data

#### 3.4.1. Quantitative Comparison of Segmentation Performance on MEF-PN35 Dataset

**Figure 3:**
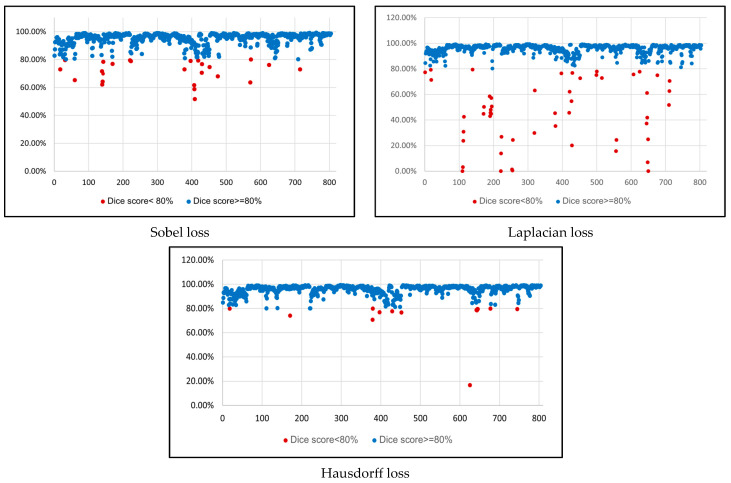
Dice similarity coefficients of 804 lung nodule patches from 35 patients at University Medical Center Ho Chi Minh City, evaluated under three boundary-aware loss functions: Sobel, Laplacian, and Hausdorff. Each dot represents a segmentation result for one patch; red dots indicate cases with suboptimal performance (Dice score < 80%). The Hausdorff configuration demonstrates the most stable and consistent performance, while the Laplacian loss yields higher variance and more failure cases.

This section quantitatively evaluates the segmentation efficacy of CAAF-ResUNet across three boundary-aware loss configurations using the MEF-PN35 dataset, which consisted of 804 CT slices obtained from 35 patients at the University Medical Center Ho Chi Minh City. The results are summarized in Table 8, while Figure 3 illustrates the patch-wise distribution of Dice scores under three different boundary-aware loss configurations.

As shown in Figure 3, the Sobel- and Hausdorff-based configurations exhibited high segmentation stability, in most cases achieving Dice scores ≥ 80%. This indicates that the model predictions are spatially well-aligned with expert annotations. High Dice scores typically occur when nodules have well-defined boundaries and are accurately localized and segmented in both shape and position. In contrast, the Laplacian-based configuration showed greater variability, with a larger number of outlier cases falling below 80%—and some even below 50%—indicating potential instability when processing heterogeneous or low-contrast nodules in clinical CT scans.

From the aggregate results in Table 9, the Hausdorff configuration yielded the highest average Dice score (95.34 ± 5.08)%, highest sensitivity (95.40 ± 6.28)%, and lowest Miss Rate (4.60 ± 6.28)%.

#### 3.4.2. Representative Cases with Segmentation Challenges on the MEF-PN35 Dataset

To complement the quantitative evaluation, we present a set of representative cases from the clinical dataset that exhibited typical segmentation challenges. These cases were selected to reflect diverse morphological characteristics observed in pulmonary nodules, such as spiculated boundaries, small size, vessel adherence, pleural attachment, cavitation, and clear isolation. Rather than aiming for exhaustive categorization, our intent is to highlight a range of practical difficulties encountered in real-world scenarios and to qualitatively assess how different boundary-aware loss functions respond to these variations. Each challenge is labeled (C1) through (C5) in Table 10 and is consistently referenced in the corresponding detailed illustrations that follow.

Following the summary in Table 11, we present detailed visual comparisons for selected representative cases, each corresponding to a specific segmentation challenge. These cases are consistently labeled using the Challenge ID (CID) codes defined in Table 9 to facilitate cross-referencing between the summary and the case illustrations. Each table reports segmentation results across three loss configurations (Sobel, Laplacian, and Hausdorff) using five columns. Loss indicates the boundary loss formulation applied in each case, where Dice loss is combined with a boundary term computed using Sobel, Laplacian, or Hausdorff operators. The Segmentation Result column shows four images side by side: the input CT patch, the manual ground truth, the model prediction, and a color-coded difference map between ground truth and prediction. In this map, green indicates true positives, blue represents true negatives, red corresponds to false positives, and yellow denotes false negatives. The final column, Attention Map, presents Grad-CAM visualizations derived from one of the Adaptive Attention Fusion (AAF) layers in the encoder path. These maps illustrate the spatial focus of the model, with red/yellow regions indicating strong attention and lighter or neutral tones indicating lower activation. All six cases are presented in detail in Table 11, Table 12 and Table 13, providing a comprehensive qualitative analysis of how each loss configuration performs under specific segmentation challenges observed in real clinical data.

## 4. Discussion

### 4.1. Learning to Align: Discussion on Loss Functions for Boundary Preservation

Precise border delineation is crucial in pulmonary nodule segmentation, since minor structural variations might result in substantial clinical implications. Although region-based measures such as Dice are commonly employed, the ability to match projected and actual boundaries—particularly in difficult scenarios—serves as a more robust measure of model efficacy. This section analyzes the impact of various boundary-aware loss settings on the model’s capacity for boundary alignment and structural preservation.

The comparative analysis of the three boundary-aware loss configurations reveals notable differences in convergence stability, boundary sensitivity, and clinical significance. While the Sobel-based loss showed rapid and consistent convergence, its dependence on first-order gradients often emphasizes local edge transitions. This trait facilitates the effective rectification of initial misalignments but may constrain the model’s capacity to discern nuanced structural attributes, especially in nodules with diffuse or low-contrast margins.

The Laplacian-based configuration, although theoretically adept at capturing higher-order edge transitions, demonstrated unstable learning behavior, particularly during the first training phases. This is probably because of the Laplacian operator’s susceptibility to intensity changes and noise, which can amplify local discrepancies in boundary predictions. The decreased Dice scores and increased performance variability, especially on the LIDC-IDRI dataset, corroborate this conclusion.

The Hausdorff-based loss, executed using the Euclidean gap Transform (EDT), prioritizes global boundary alignment by imposing penalties on the largest gap between predicted and ground truth contours. Despite the training loss values being comparatively elevated, this indicates a more rigorous evaluation standard rather than inadequate optimization. This configuration achieved the greatest Dice score of 90.88% and the lowest Miss Rate of 7.62%, indicating that global consistency is essential for enhancing segmentation robustness.

In addition, the attention behavior learned under Hausdorff supervision provides further insights. The AAC consistently emphasized channel attention (w_2_) over position attention (w_1_), indicating that when guided by global boundary constraints, the model learns to prioritize semantic continuity over fine-grained spatial localization. This behavior aligns with the clinical need to preserve anatomical structure rather than overfit to noisy local gradients.

### 4.2. Adaptive Attention for Contextual Feature Fusion

The performance gains observed with AAC integration underscore the effectiveness of data-driven attention fusion strategies in medical image segmentation. Static weighting methods are inherently limited in their ability to adapt to local or global variations in nodule presentation, whereas AAC enables the network to dynamically adjust the balance between spatial and semantic cues based on contextual demands.

This adaptability can be partially attributed to the architecture’s ability to capture relational information among spatial locations via self-attention, followed by global summarization through spatial averaging. The resulting global feature representation informs the generation of adaptive weights through a nonlinear transformation using fully connected layers. This design allows the controller to weigh the importance of spatial layout versus semantic content, depending on the structural complexity of the input.

Interestingly, across all boundary-aware loss configurations—including edge-focused and global alignment strategies—the AAC consistently favored semantic-level aggregation, as reflected in the higher values of channel attention weights (w_2_). This suggests that, regardless of the specific formulation of the boundary loss, the model learned to prioritize semantic continuity when boundary information was introduced. Such behavior aligns well with clinical expectations, where preserving anatomical structure is often more important than optimizing for localized edge precision.

In this light, AAC not only improves segmentation accuracy but also enhances the model’s capacity to reason over context, allowing it to adapt its fusion behavior in a way that is both data-specific and clinically meaningful.

### 4.3. Toward Clinical Readiness: From Benchmark Comparison to Real-World Validation of CAAF-ResUNet

The performance of CAAF-ResUNet, as benchmarked against existing state-of-the-art segmentation models in Table 7, demonstrates clear architectural advantages that directly translate to higher segmentation quality. Prior works such as U-Det, the Bidirectional Feature Network, and the Dual Encoding Fusion Network have made significant progress through encoder–decoder refinements and multi-branch architectures. However, these models largely rely on static attention mechanisms or handcrafted fusion strategies, which limit their ability to adapt to the wide variability found in pulmonary nodules—especially in clinical imaging.

In contrast, CAAF-ResUNet introduces a data-driven attention fusion strategy through the Adaptive Attention Controller (AAC), which dynamically adjusts the balance between channel and position attention based on contextual features. This enables the model to generalize more effectively across diverse anatomical presentations, as evidenced by its superior Dice scores and lower miss rates across both LUNA16 and LIDC-IDRI datasets.

To further assess the model’s readiness for clinical deployment, we evaluated its performance on a real-world dataset comprising 804 CT slices from 35 patients at the University Medical Center Ho Chi Minh City. As shown in Table 8, the model achieved a Dice score of (95.34 ± 5.08)%, sensitivity of (95.40 ± 6.28)%, and Miss Rate of (4.60 ± 6.28)% using the Hausdorff loss configuration. The detailed results for each individual case are presented in Appendix A.1.

These metrics collectively confirm the model’s ability to accurately and comprehensively delineate pulmonary nodules. A high Dice score indicates precise spatial coverage and boundary alignment, while high sensitivity reflects the model’s effectiveness in detecting even subtle or ambiguous nodules. Crucially, the low Miss Rate highlights the model’s ability to avoid false negatives—an essential requirement in early lung cancer screening, where diagnostic oversight can lead to delayed treatment and poorer outcomes.

Beyond quantitative metrics, the model has also demonstrated strong performance across a diverse range of challenging clinical scenarios. As detailed in Table 11, Table 12 and Table 13, CAAF-ResUNet effectively handled representative cases including nodules with clear boundaries (C1), small size (C2), blurred margins (C3a–C3b), cavitary structures (C4), and spiculated edges (C5). These examples underscore the model’s ability to adapt to a wide spectrum of morphological complexities often encountered in routine CT interpretation.

Collectively, these results demonstrate that CAAF-ResUNet is not only architecturally and algorithmically superior to prior models but also clinically ready—capable of handling real-world data variability with high precision and minimal diagnostic risk.

## 5. Conclusions

This study introduces CAAF-ResUNet, a segmentation framework integrating Context-Aware Adaptive Attention Fusion (CAAF) with an improved Adaptive Attention Controller (AAC) to enhance lung nodule segmentation in CT scans. The model effectively addresses challenges in nodule boundary delineation, small-scale object segmentation, and feature prioritization through an advanced attention fusion strategy combining channel and position attention. The proposed integration of Dice loss with boundary-sensitive loss functions (Sobel, Laplacian, Hausdorff) significantly improves segmentation accuracy. Dice + Hausdorff loss demonstrated superior performance, achieving a Dice score up to 90.88% on LUNA16 and 85.92% on LIDC-IDRI, with a notable reduction in the Miss Rate. The introduction of AAC further refines feature weighting, dynamically adjusting the balance between channel and position attention. Experimental results confirm that incorporating AAC enhances segmentation robustness, increasing Dice scores on LUNA16 and LIDC-IDRI compared to models without AAC.

The model was validated on 35 real-world patient cases from the University Medical Center Ho Chi Minh City, achieving an average Dice score of 95.34% and a Miss Rate of 4.60%, demonstrating high generalizability in clinical settings. Compared to existing segmentation architectures, CAAF-ResUNet surpasses U-Net, Res-UNet, and Attention U-Net, achieving the highest Dice, IoU, and sensitivity scores, while maintaining a lower Miss Rate, reinforcing its effectiveness in segmenting ambiguous lung nodules. The proposed context-aware adaptive fusion mechanism enhances segmentation performance, particularly in challenging cases where nodule boundaries are indistinct.

These findings highlight the importance of attention-based fusion and boundary-aware loss functions in medical image segmentation. The proposed framework has the potential to be extended to other medical imaging applications, including tumor segmentation in MRI scans and pathology detection in histopathological images.

## Figures and Tables

**Table 1:** Summary of key research gaps in different categories of segmentation models, highlighting their strengths and weaknesses.

Model Category	Example Models	Common Features	Challenges
U-Net-based architectures	U-Net, U-Net++, ResUNet	Encoder–decoder structure with skip connections; preserves spatial features.	Struggles with boundary refinement and precise feature selection, leading to blurry segmentation contours.
Attention-based models	Attention U-Net, DANet	Introduces attention mechanisms to refine feature selection.	Fixed attention weight distributions limit adaptability to different nodule characteristics.
Transformer-based models	TransUNet, Swin-UNet, MCAT-Net	Captures long-range dependencies using self-attention mechanisms.	Computationally expensive, making real-time clinical applications challenging.

**Table 2:** Computational complexity of adaptive attention controller.

Operation	Complexity
Adaptive Average Pooling	*O (B* × *C* × *H* × *W)*
Linear projections (*Q, K, V*)	*O (B* × *N* × *C^2^)*
Attention computation	*O (B* × *N*^2^ × *C)*
Attention-weighted sum	*O (B* × *N*^2^ × *C)*
Spatial averaging	*O (B* × *N* × *C)*
Fully connected layers	*O (B* × *C*^2^*)*

**Table 3:** Several key benefits of AAF.

Fusion Method	Weight Assignment	Context Awareness	Adaptability
Fixed Weights	Manually assigned	No	Low
Static Attention Fusion	Equal weights for CA and PA	Partial	Medium
Adaptive Attention Fusion (AAF) (Ours)	Learned dynamically via AAC	Yes	High

**Table 4:** Number of training and testing samples in LUNA16, LIDC, and MEF-PN35 datasets.

Dataset	Train	Test
LIDC-IDRI	13.928	1.200
LUNA16	9.800	1.200
MEF-PN35		804

**Table 5:** Quantitative segmentation results of the CAAF-ResUNet-trained boundary-aware losses on LUNA16 and LIDC-IDRI datasets. Metrics include Dice score, Intersection over Union (IoU), Sensitivity, Miss Rate, and specificity (mean ± standard deviation).

Dataset	Loss Operator	Dice Score (%)	IoU (%)	Sensitivity (%)	Miss Rate (%)	Specificity (%)	Mean w_1_	Mean w_2_
LUNA16	Sobel	88.97 ± 9.33	81.22 ± 13.07	89.63 ± 11.96	10.37 ± 11.96	99.46 ± 0.98	0.27	0.73
	Laplacian	85.37 ± 22.20	78.99 ± 23.56	87.30 ± 22.13	12.70 ± 22.13	99.44 ± 0.90	0.36	0.64
	Hausdorff	90.88 ± 6.16	83.83 ± 9.88	91.08 ± 10.20	8.92 ± 10.20	99.49 ± 1.06	0.26	0.74
LIDC-IDRI	Sobel	85.67 ± 16.96	77.93 ± 20.58	91.33 ± 17.63	8.67 ± 17.63	99.38 ± 0.99	0.24	0.76
	Laplacian	85.33 ± 17.86	77.62 ± 21.07	90.31 ± 18.41	9.69 ± 18.41	99.31 ± 1.23	0.31	0.69
	Hausdorff	85.92 ± 16.70	78.24 ± 20.37	92.38 ± 16.88	7.62 ± 16.88	99.28 ± 1.18	0.22	0.78

**Table 6:** Statistical comparison between AAC and non-AAC model variants under different boundary-aware loss functions using paired *t*-tests on Dice scores across 1200 test cases from LUNA16.

Loss Function	AAC	Dice Score (%)	t-Statistic	*p*-Value
Dice + Sobel	No	87.46 ± 7.32	5.98	2.74 × 10^−9^
	Yes	88.97 ± 9.33		
Dice + Laplacian	No	84.14 ± 16.90	2.38	0.017
	Yes	85.37 ± 22.20		
Dice + Hausdorff	No	88.40 ± 7.04	15.91	6.18 × 10^−52^
	Yes	90.88 ± 6.16		

**Table 7:** Segmentation performance of CAAF-ResUNet with and without the Adaptive Attention Controller (AAC), compared against fixed-weight attention configurations and the ResUNet++ baseline on LUNA16 and LIDC-IDRI datasets. Results are reported as mean Dice score (%), demonstrating the effectiveness of dynamic attention fusion.

Dataset	Model	AAC	w_1_	w_2_	Dice Score (%)
LUNA16	ResUnet++ (baseline)	No			83.89
	Position + ResUnet	No			82.79
	Channel + ResUnet	No			84.44
	PCAM + ResUnet	No	0.5	0.5	85.96
	CAAF-ResUnet (Hausdorff)	Yes	0.26	0.74	90.88
LIDC-IDRI	PCAM + ResUnet	No	0.5	0.5	85.37
	CAAF-ResUnet (Hausdorff)	Yes	0.22	0.78	85.92

**Table 9:** Segmentation performance of CAAF-ResUNet on real-world cases provided by the University Medical Center Ho Chi Minh City.

Loss Function	Dice Score (%)	IoU (%)	Sensitivity (%)	Miss Rate (%)	Specificity (%)	Mean w_1_	Mean w_2_
Sobel	94.69 ± 5.81	90.42 ± 9.13	94.00 ± 7.37	6.00 ± 7.37	98.87 ± 1.23	0.26	0.74
Laplacian	92.54 ± 14.38	88.31 ± 16.60	93.16 ± 13.53	6.84 ± 13.53	98.30 ± 2.33	0.31	0.69
Hausdorff	95.34 ± 5.08	91.47 ± 7.74	95.40 ± 6.28	4.60 ± 6.28	98.64 ± 1.76	0.21	0.79

**Table 10:** Representative segmentation challenges in pulmonary nodules, categorized by morphological characteristics. Each row includes a CT slice and cropped nodule patch with corresponding diameter, illustrating key clinical and algorithmic difficulties such as boundary clarity and nodule size.

Segmentation Challenge	Key Morphological Characteristics	Illustrative Slice	Nodule Patch (Diameter, mm)
Clearly defined boundary (C1)	Isolated nodules, surrounded by homogeneous lung parenchyma with clearly defined margins, provide strong boundary contrast and minimal anatomical ambiguity, facilitating high segmentation accuracy.	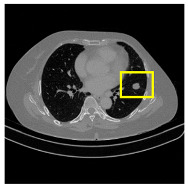	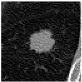 (14.83)
Small size (C2)	Small nodules (<5 mm) often appear morphologically similar to blood vessels or imaging noise, posing significant challenges for detection and leading to high false negative rates in segmentation.	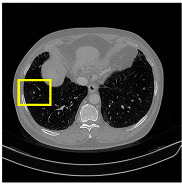	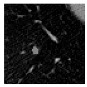 (4.74)
Blurred boundary (C3)	Nodule in direct contact with blood vessels or the chest wall, leading to unclear margins due to poor contrast and anatomical integration. This constrains the model’s capacity to precisely differentiate the lesion from surrounding components.	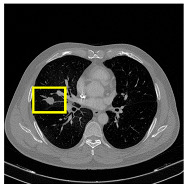	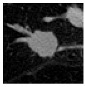 (19.79)
		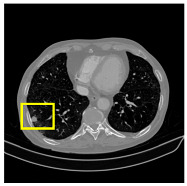	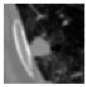 (12.38)
Heterogeneous core (C4)	Cavitary nodules contain internal low-density regions, leading to intra-lesion intensity variation that complicates consistent mask prediction and increases segmentation uncertainty.	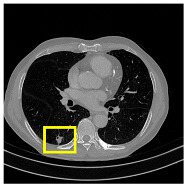	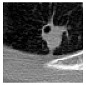 (16.45)
Irregular boundary (C5)	Spiculated nodules exhibit irregular and radiating extensions that blend with surrounding tissue, causing boundary fragmentation and frequent false negatives in segmentation maps.	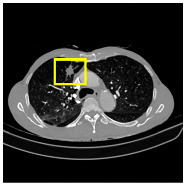	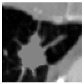 (25.31)

**Table 11:** Segmentation results for C1 (clear boundary) and C2 (small nodule).

CID	Loss Function	Dice Score (%)	Segmentation Result	Attention Map
C1	Sobel	93.85	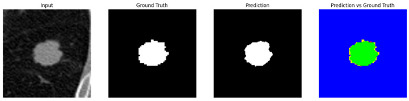	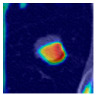
	Laplacian	93.09	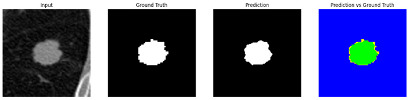	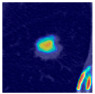
	Hausdorff	92.80	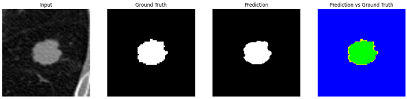	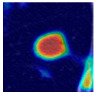
C2	Sobel	73.97	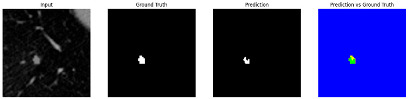	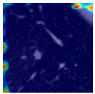
	Laplacian	44.79	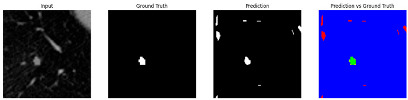	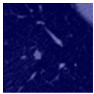
	Hausdorff	91.95	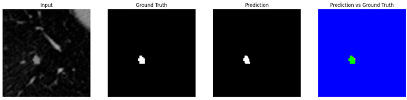	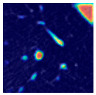

**Table 12:** Segmentation results for C3a (vessel adherence) and C3b (chest wall attachment).

CID	Loss Function	Dice Score (%)	Segmentation Result	Attention Map
C3a	Sobel	96.29	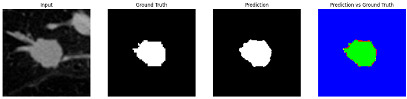	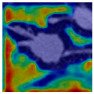
	Laplacian	96.37	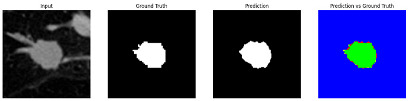	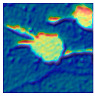
	Hausdorff	96.59	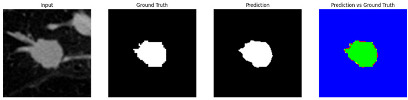	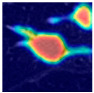
C3b	Sobel	91.77	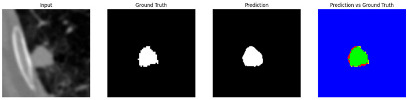	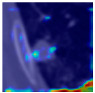
	Laplacian	92.82	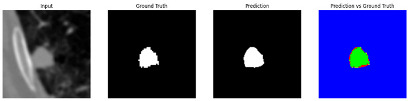	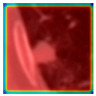
	Hausdorff	93.21	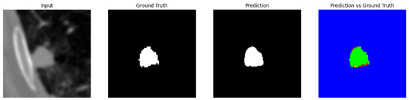	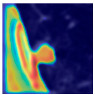

**Table 13:** Segmentation results for C4 (cavitary nodule) and C5 (spiculated nodule).

CID	Loss Function	Dice Score (%)	Segmentation Result	Attention Map
C4	Sobel	89.62	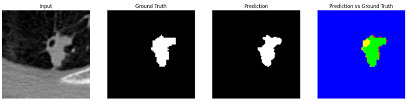	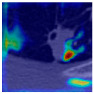
	Laplacian	91.67	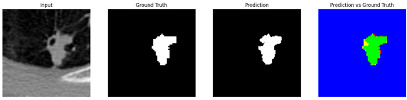	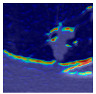
	Hausdorff	89.63	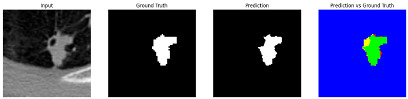	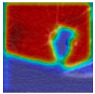
C5	Sobel	97.45	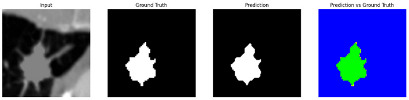	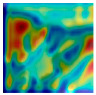
	Laplacian	97.18	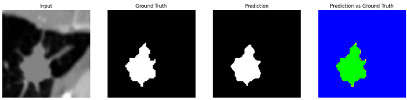	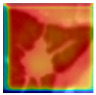
	Hausdorff	97.49	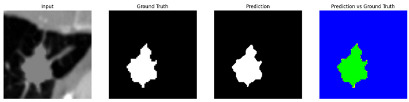	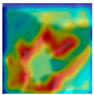

## Data Availability

The datasets generated and analyzed during the current study are not publicly available due to institutional restrictions and patient confidentiality agreements. However, the data may be made available from the corresponding author upon reasonable request and with permission from the University Medical Center Ho Chi Minh City.

## Abbreviations

The following abbreviations are used in this manuscript:

AAF	Adaptive Attention Fusion
AAC	Adaptive Attention Controller
ASPP	Atrous Spatial Pyramid Pooling
CA	Channel Attention
CNN	Convolutional Neural Network
CT	Computed Tomography
FCN	Fully Connected Network
FN	False Negative
FP	False Positive
GAP	Global Average Pooling
GMP	Global Max Pooling
IoU	Intersection over Union
K	Key
LIDC-IDRI	Lung Image Database Consortium and Image Database Resource Initiative
LUNA16	Lung Nodule Analysis 2016
PA	Position Attention
Q	Query
TN	True Negative
TP	True Positive
V	Value

## Appendix A

### Appendix A.1

Table A1 presents the detailed segmentation results across 35 anonymized patient cases, indexed from Case 1 to Case 35. For each case, we report the number of lung-nodule-containing patches extracted, with a total of 804 patches evaluated. The table summarizes the average performance metrics per case, including Dice score, Intersection over Union (IoU), sensitivity, Miss Rate, and specificity, providing a case-wise view of the model’s segmentation consistency on real-world CT data.

**Table A1:** Per-case segmentation performance metrics of CAAF-ResUNet on anonymized CT scans from 35 patients. Each case includes the number of annotated lung-nodule patches and the corresponding average scores across key evaluation metrics (Dice, IoU, sensitivity, Miss Rate, and specificity).

Case	No. of Patches	Mean Dice Score (%)	Mean IoU (%)	Mean Sensitivity (%)	Mean Miss Rate (%)	Mean Specificity (%)
1	19	91.26%	84.30%	91.36%	8.64%	98.60%
2	14	90.23%	82.35%	87.46%	12.54%	99.33%
3	34	97.60%	95.34%	97.24%	2.76%	99.48%
4	15	96.53%	93.33%	96.75%	3.25%	99.23%
5	30	93.63%	88.33%	92.20%	7.80%	98.82%
6	31	97.08%	94.35%	97.03%	2.97%	98.56%
7	24	96.49%	93.57%	96.23%	3.77%	99.18%
8	26	98.06%	96.20%	97.62%	2.38%	99.49%
9	21	91.39%	84.42%	92.07%	7.93%	97.64%
10	12	96.47%	93.19%	95.73%	4.27%	99.69%
11	32	96.64%	93.58%	96.27%	3.73%	98.93%
12	33	97.88%	95.87%	97.97%	2.03%	99.29%
13	16	96.61%	93.48%	96.89%	3.11%	98.62%
14	30	97.28%	94.74%	97.71%	2.29%	98.26%
15	14	95.06%	90.67%	95.13%	4.87%	99.00%
16	19	91.10%	84.35%	90.48%	9.52%	98.59%
17	12	91.88%	85.06%	91.06%	8.94%	98.59%
18	10	85.33%	74.49%	83.29%	16.71%	99.41%
19	9	97.28%	94.77%	97.18%	2.82%	99.81%
20	24	87.39%	77.90%	85.53%	14.47%	98.15%
21	23	97.98%	96.07%	97.99%	2.01%	99.32%
22	23	97.61%	95.34%	97.12%	2.88%	99.22%
23	18	96.73%	93.71%	96.93%	3.07%	99.25%
24	27	95.34%	91.47%	95.40%	4.60%	98.64%
25	23	97.56%	95.26%	97.55%	2.44%	99.44%
26	16	94.97%	90.49%	94.79%	5.21%	99.38%
27	40	97.35%	94.88%	96.97%	3.03%	99.57%
28	30	94.78%	92.22%	94.56%	5.44%	98.87%
29	25	89.97%	82.29%	96.72%	3.28%	96.36%
30	26	97.63%	95.39%	97.99%	2.01%	98.84%
31	14	92.04%	85.72%	94.30%	5.70%	91.32%
32	21	97.14%	94.46%	96.77%	3.23%	98.66%
33	33	97.37%	94.91%	98.18%	1.82%	96.94%
34	30	95.74%	92.21%	97.70%	2.30%	98.61%
35	30	97.71%	95.60%	98.26%	1.74%	98.69%
	804	95.34%	91.47%	95.40%	4.60%	98.64%
